# LncRNA *miR663AHG* represses the development of colon cancer in a *miR663a*-dependent manner

**DOI:** 10.1038/s41420-023-01510-1

**Published:** 2023-07-03

**Authors:** Hongfan Yuan, Qianwen Ren, Yantao Du, Yuwan Ma, Liankun Gu, Jing Zhou, Wei Tian, Dajun Deng

**Affiliations:** 1 Key Laboratory of Carcinogenesis and Translational Research (MOE/Beijing), Division of Cancer Etiology, Peking University Cancer Hospital and Institute, Beijing, 100142 China; 2 The Department of Medical Oncology, Sichuan Cancer Hospital and Institute, Affiliated Cancer Hospital of University of Electronic and Technology of China, Chengdu, 610042 China; 3 The First Affiliated Hospital of Ningbo University, Ningbo, Zhejiang 315010 China; 4 Collaborative Innovation Center for Cancer Personalized Medicine, Nanjing Medical University, Nanjing, 211166 China

**Keywords:** Oncogenesis, Oncogenes

## Abstract

The *MIR663AHG* gene encodes both *miR663AHG* and *miR663a*. While *miR663a* contributes to the defense of host cells against inflammation and inhibits colon cancer development, the biological function of lncRNA *miR663AHG* has not been previously reported. In this study, the subcellular localization of lncRNA *miR663AHG* was determined by RNA-FISH. *miR663AHG* and *miR663a* were measured by qRT-PCR. The effects of *miR663AHG* on the growth and metastasis of colon cancer cells were investigated in vitro and in vivo. CRISPR/Cas9, RNA pulldown, and other biological assays were used to explore the underlying mechanism of *miR663AHG*. We found that *miR663AHG* was mainly distributed in the nucleus of Caco2 and HCT116 cells and the cytoplasm of SW480 cells. The expression level of *miR663AHG* was positively correlated with the level of *miR663a* (*r* = 0.179, *P* = 0.015) and significantly downregulated in colon cancer tissues relative to paired normal tissues from 119 patients (*P* < 0.008). Colon cancers with low *miR663AHG* expression were associated with advanced pTNM stage (*P* = 0.021), lymph metastasis (*P* = 0.041), and shorter overall survival (hazard ratio = 2.026; *P* = 0.021). Experimentally, *miR663AHG* inhibited colon cancer cell proliferation, migration, and invasion. The growth of xenografts from RKO cells overexpressing *miR663AHG* was slower than that of xenografts from vector control cells in BALB/c nude mice (*P* = 0.007). Interestingly, either RNA-interfering or resveratrol-inducing expression changes of *miR663AHG* or *miR663a* can trigger negative feedback regulation of transcription of the *MIR663AHG* gene. Mechanistically, *miR663AHG* could bind to *miR663a* and its precursor *pre-miR663a*, and prevent the degradation of *miR663a* target mRNAs. Disruption of the negative feedback by knockout of the *MIR663AHG* promoter, exon-1, and *pri-miR663A*-coding sequence entirely blocked these effects of *miR663AHG*, which was restored in cells transfected with *miR663a* expression vector in rescue experiment. In conclusion, *miR663AHG* functions as a tumor suppressor that inhibits the development of colon cancer through its *cis*-binding to *miR663a/pre-miR663a*. The cross talk between *miR663AHG* and *miR663a* expression may play dominant roles in maintaining the functions of *miR663AHG* in colon cancer development.

## Introduction

Colon cancer is a malignant disease with a mortality rate second to that of lung cancer worldwide. In 2022, it is estimated that there will be ~100,000 newly diagnosed colon cancer patients in the United States, and the mortality rate will reach ~8.6% [1]. Although the popularization of DNA methylation marker screening, colonoscopy examination, and resection of colorectal mucosal polyps have made considerable progress in early diagnosis and prevention, the incidence of colon cancer in people under 50 years old is still steadily increasing [1]. In-depth investigations of the mechanisms may hasten elucidation of the development of colon cancer.

With the development of high throughput RNA sequencing (RNA-seq) technology, it has been found that there are a large number of noncoding RNA (ncRNA) molecules transcribed from the human genome [2]. Long noncoding RNAs (lncRNAs) may regulate chromatin conformation, gene transcription, hnRNA splicing, and other biological processes such as cancer development [3–8]. The expression status of many lncRNAs is abnormal in cancer tissues. Some factors directly affect cancer cell proliferation, apoptosis, metastasis, angiogenesis, drug resistance, and stem cell stemness [9–12]. In addition, ncRNAs have tissue-specific and tumor-specific expression patterns and are expected to be potential screening, diagnostic, and prognostic biomarkers for colorectal cancer in stool, serum/plasma, and tissue samples of patients [13–15].

*MIR663AHG* is a rare human-specific gene (Entrez Gene: 284801) located in the centromere of chromosome 20. The primary transcript (hnRNA) of this gene can be simultaneously spliced into microRNA *miR663a* and lncRNA *miR663AHG*. We have previously reported that *miR663a* expression is downregulated in colon cancer tissues and that *miR663a* inhibits the growth and metastasis of colon cancer cells [7, 16]. A correlation investigation suggests that changes in *miR663AHG* expression are associated with the occurrence of intervertebral disc degeneration (IDD) [17]. Another study revealed that *miR663AHG* is downregulated in psoriatic tissues [18]. According to the Genotype-Tissue Expression Project (GTEx) project database [19], *miR663AHG* is ubiquitously expressed in various human normal tissues with relative abundance (Fig. S1). However, the biological functions of *miR663AHG* have not been previously reported. In this study, we studied, for the first time, the roles of *miR663AHG* in colon cancer development and its molecular mechanisms.

## Results

### Characterization of *miR663AHG* and its subcellular distribution

**Fig. 1:**
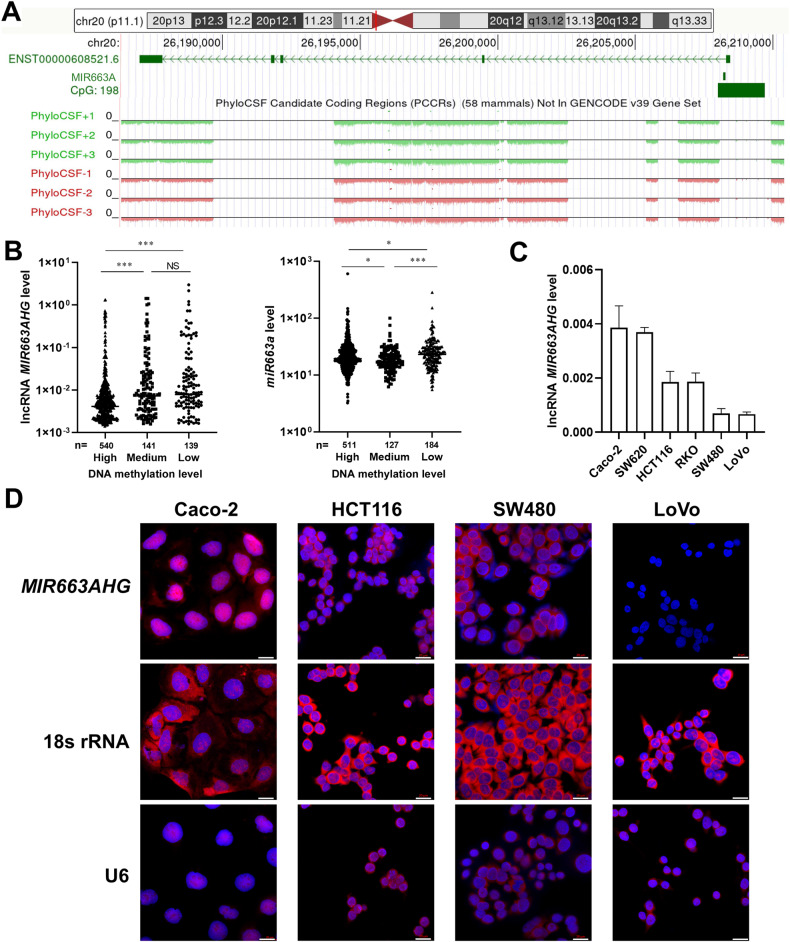
Characterization of lncRNA *miR663AHG*. **A** The prediction of the protein-coding ability of *miR663AHG*, as analyzed with the PhyloCSF online tool. The genomic locations of *miR663AHG* exons, *miR663a*, and CpG islands in human chromosome 20 are also illustrated using the adapted images downloaded from the UCSC website. **B** Comparisons of the levels of *miR663AHG* and *miR663a* in cancer cell lines subclassified into *MIR663AHG* DNA methylation-high, -moderate, and -low groups according to the CCLE databases. */***: *P* < 0.05/0.001 and NS: *P* > 0.05 in Mann–Whitney U-test. **C** The baseline level of *miR663AHG* expression in six colon cancer cell lines, as determined by qRT-PCR. **D** Subcellular distribution of endogenous *miR663AHG* in various colon cancer cells in the RNA-FISH analysis; *18* *S rRNA* and *U6* were used as cytoplasmic and nuclear RNA controls, respectively.

Compared with protein-coding genes, the transcription start sites (TSSs) of ncRNA genes are more diverse [20, 21]. *MIR663AHG* is a multiexon gene. *miR663a* and *miR663AHG* are spliced from the same hnRNA. The values of *miR663AHG* were less than zero in PhyloCSF analysis (Fig. 1A), suggesting that this lncRNA does not encode a protein [22].

It is well-known that gene transcription is regulated by the methylation status of CpG islands around transcription start sites (TSSs). A CpG island is located around the *MIR663AHG* TSS (Fig. 1A). According to the results of mining CCLE DNA methylation and RNA-seq databases [23], the levels of *miR663AHG* and *miR663a* were significantly lower in cell lines with high methylation levels than in those with moderate or low methylation levels (*P* < 0.05; Fig. 1B). This suggests that the transcription of the *MIR663AHG* gene is regulated by DNA methylation.

In eukaryotic cells, the functions of lncRNAs are closely related to their subcellular localization. To characterize the distribution pattern of *miR663AHG* in colon cancer cells, its baseline expression status in six colon cancer cell lines was initially determined by qRT-PCR. The level of *miR663AHG* was higher in Caco2 or SW620 cells than in HCT116 or RKO cells and lowest in SW480 and LoVo cells (Fig. 1C). Similarly, the level of *miR663a* was highest in SW620 cells and lowest in SW480 cells (Data not shown). The results of RNA-FISH analysis showed that *miR663AHG* was located in both the cytoplasm and nucleus of colon cancer cells: mainly in the nucleus of Caco2 and HCT116 cells and in the cytoplasm of SW480 cells (Fig. 1D). No *miR663AHG* hybridization signal could be detected in LoVo cells.

### Downregulation of *miR663AHG* in colon cancer is associated with poor prognosis

**Fig. 2:**
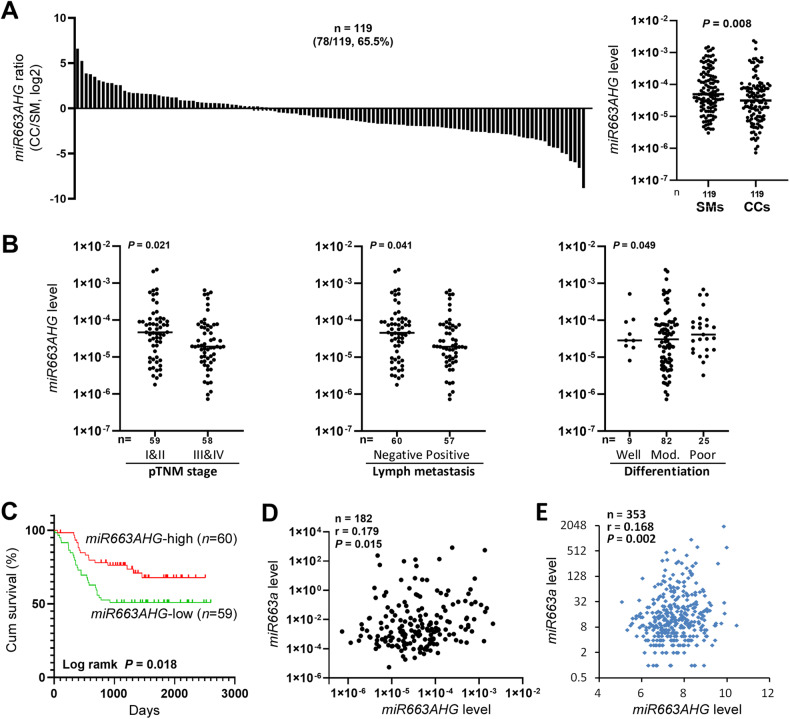
The level of *miR663AHG* expression in colon cancer tissue samples from patients with various clinicopathological characteristics. **A** The level of *miR663AHG* expression in colon cancer (CC) and paired surgical margin (SM) samples from 119 patients, as measured by qRT-PCR. The *P-*value in the Mann–Whitney U test and the ratio of CCs with *miR663AHG* downregulation are labeled. **B** Comparisons of the level of *miR663AHG* expression in CCs at different pTNM stages, lymph metastasis, and differentiation states. Detailed information is listed in Table S1. **C** Overall survival curves for patients with colon cancer with high and low *miR663AHG* expression (according to the *median* value). **D** The coexpression status of *miR663AHG* and *miR663a* in 182 (CC and SM) tissue samples. **E** The coexpression status of *miR663AHG* and *miR663a* in 353 ovarian cancer tissue samples. The Spearman correlation coefficient (*r*) is labeled.

We previously reported that *miR663a* expression was significantly downregulated in colon cancer tissues (CCs) relative to matched surgical margin controls (SMs) from patients [7]. Patients (*n* = 119) for whom enough RNA samples were available for *miR663AHG* detection were re-enrolled in the present study. The results of the qRT-PCR analysis showed that *miR663AHG* expression was significantly downregulated in CCs compared with SMs, especially in CCs with lymph metastasis or at advanced pTNM stages or with good differentiation (Fig. 2A, B; Table S1).

In the Kaplan-Meier analysis, the overall survival (OS) of patients with colon cancer with low *miR663AHG* expression (below the *median*) was significantly shorter than that of patients with high *miR663AHG* expression (*P* = 0.018; Fig. 2C). Univariate analysis showed that four risk factors were significantly associated with the OS of these patients (Table S3). These factors included low *miR663 AHG* expression (hazard ratio [HR], 2.026; 95% confidence interval [CI], 1.113–3.689), pTNM stage, lymph metastasis, and distant metastasis. In multivariate analysis, however, only the metastasis status, but not the *miR663AHG* level, was significantly associated with patients’ OS.

In addition, combined with our previously reported *miR663a* data [7], we analyzed the correlation between the levels of *miR663AHG* and *miR663a* in these colon tissues (*n* = 182; including CCs and SMs). A positive correlation was observed (*r* = 0.179, *P* = 0.015), implying a possible role of *miR663a* in the biological function of *miR663AHG* (Fig. 2D). Furthermore, we also analyzed the correlation using public microarray datasets [24] and found a similar correlation among 353 ovarian cancer samples (Spearman *r* = 0.168, *P* = 0.002; Fig. 2E).

### *miR663AHG* inhibits the growth and metastasis of colon cancer cells in vitro and in vivo

**Fig. 3:**
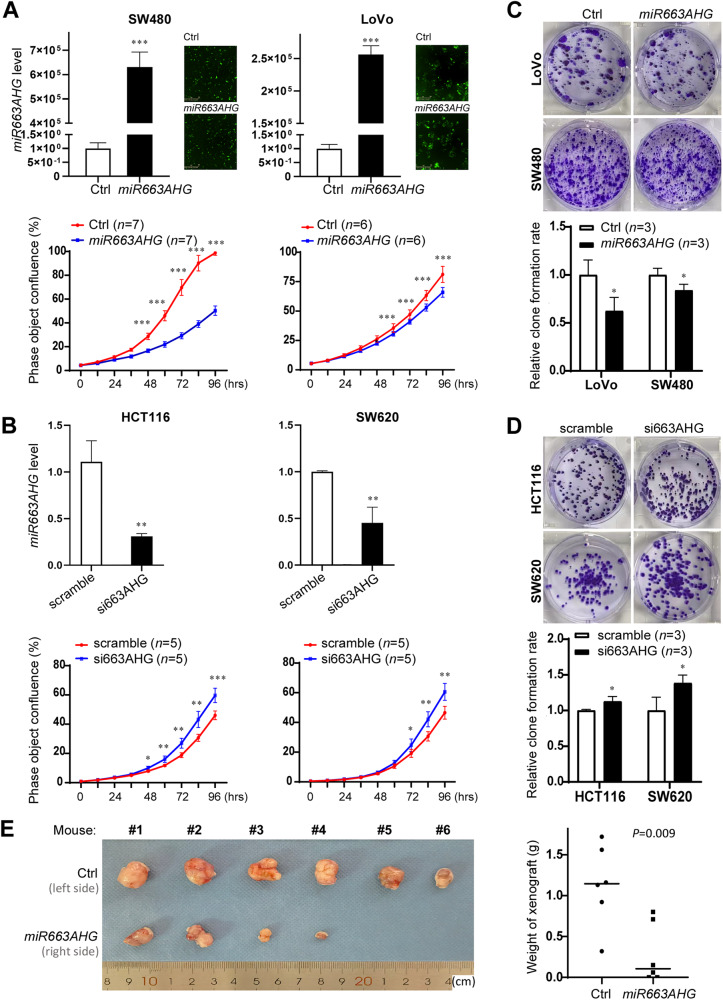
Effect of *miR663AHG* on the proliferation and growth of colon cancer cells in vitro and in vivo. **A**, **B** Impact of *miR663AHG* overexpression or knockdown on the proliferation of colon cancer cells, as measured by long-term dynamic observation. The transfection efficiency of the *miR663AHG* expression vector was monitored by examination of the level of ZsGreen expression 48 h post-transfection. The level of *miR663AHG* is presented as the mean ± SD. **C**, **D** Effect of *miR663AHG* overexpression or knockdown on the colony formation of colon cancer cells. **E** Images and tumor weights of subcutaneous xenografts derived from *miR663AHG* stably overexpressing RKO cells in BALB/c nude mice. */**/***: *P* < 0.05/0.01/0.001 for (**A**–**D**) by Student’s t-test, for (**E**) by the Mann–Whitney U-test.

To investigate the biological functions of *miR663AHG*, two human colon cancer cell lines, SW480 and LoVo, with a low baseline level of *miR663AHG* expression, were stably transfected with the lentiviral *miR663AHG* expression vector, while the other two cell lines SW620 and HCT116, with a high baseline level of *miR663AHG* expression, were transfected with siRNAs against *miR663AHG* (si663AHG). The proliferation of SW480 and LoVo cells was significantly inhibited by *miR663AHG* overexpression, whereas the proliferation of SW620 and HCT116 cells was significantly promoted by *miR663AHG* knockdown in the long-term dynamic observation analysis (Fig. 3A, B). Similar differences in the colony formation rate were also induced by both *miR663AHG* overexpression and knockdown (Fig. 3C, D). These results were confirmed in the animal model. The growth of xenografts derived from RKO cells stably overexpressing *miR663AHG* was significantly slower than that of empty control cells (*P* = 0.009; Fig. 3E).

**Fig. 4:**
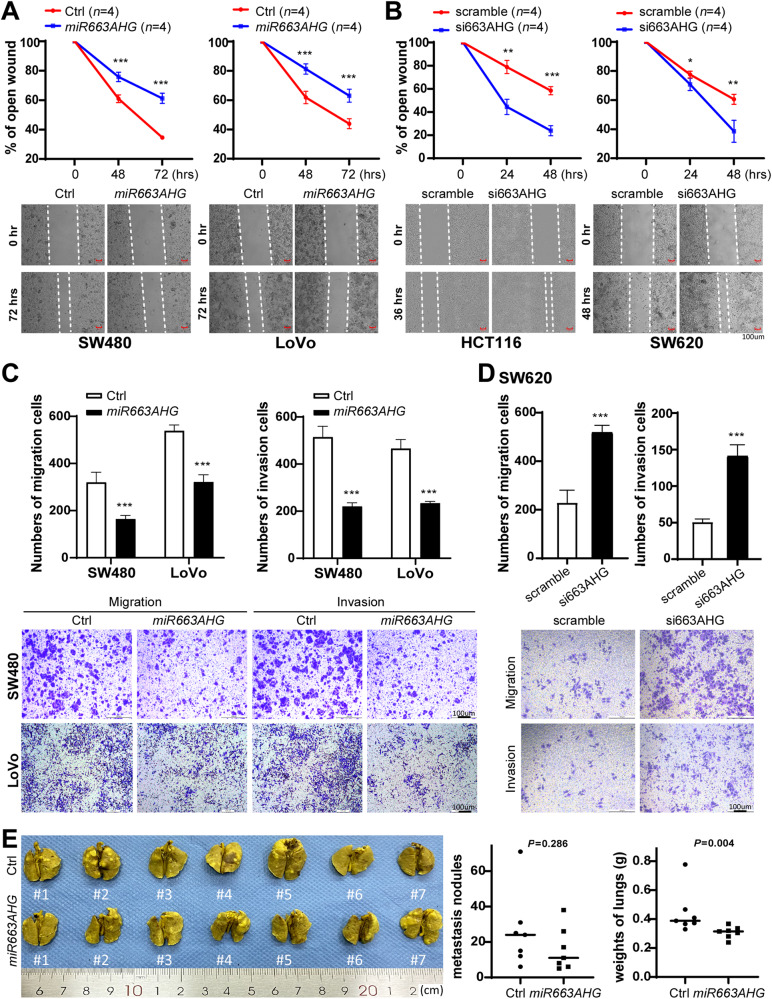
Effects of *miR663AHG* on cell migration/invasion and lung metastasis of colon cancer cells in vitro and in vivo. **A**, **B** Effects of *miR663AHG* overexpression or siRNA knockdown on the migration of colon cancer cells in wound-healing analysis. **C**, **D** Effects of *miR663AHG* overexpression or siRNA knockdown on the migration and invasion of colon cancer cells in transwell analyses. (**E**) Effect of *miR663AHG* overexpression on the experimental lung metastasis of LoVo cells in NOD/SCID mice. Both the average number of metastatic lesions and lung weight are displayed. */**/***: *P* < 0.05/0.01/0.001 for (**A**–**D**) by Student’s t-test, for (**E**) by the Mann–Whitney U-test.

Furthermore, the results of wound healing assays revealed that the migration of SW480 and LoVo cells was impeded by stable *miR663AHG* overexpression (Fig. 4A), while that of SW620 and HCT116 cells was enhanced by *miR663AHG* knockdown (Fig. 4B). The results of transwell assays also showed that *miR663AHG* overexpression or knockdown significantly decreased or increased the migration and invasion of these cells, respectively (Fig. 4C, D). Although the difference in the average number of lung surface metastatic nodules was not statistically significant (Fig. 4E left, *P* = 0.286; Fig. S3), a significant decrease in the weight of lungs was observed between NOD-SCID mice injected with LoVo cells with and without stable *miR663AHG* overexpression (*P* = 0.004; Fig. 4E right).

Collectively, these results indicate that *miR663AHG* suppresses colon cancer cell proliferation, migration, and invasion in vitro and suppresses tumor formation and lung metastasis in vivo.

### Negative feedback of the *MIR663AHG* gene by its *miR663AHG* and *miR663a* products

**Fig. 5:**
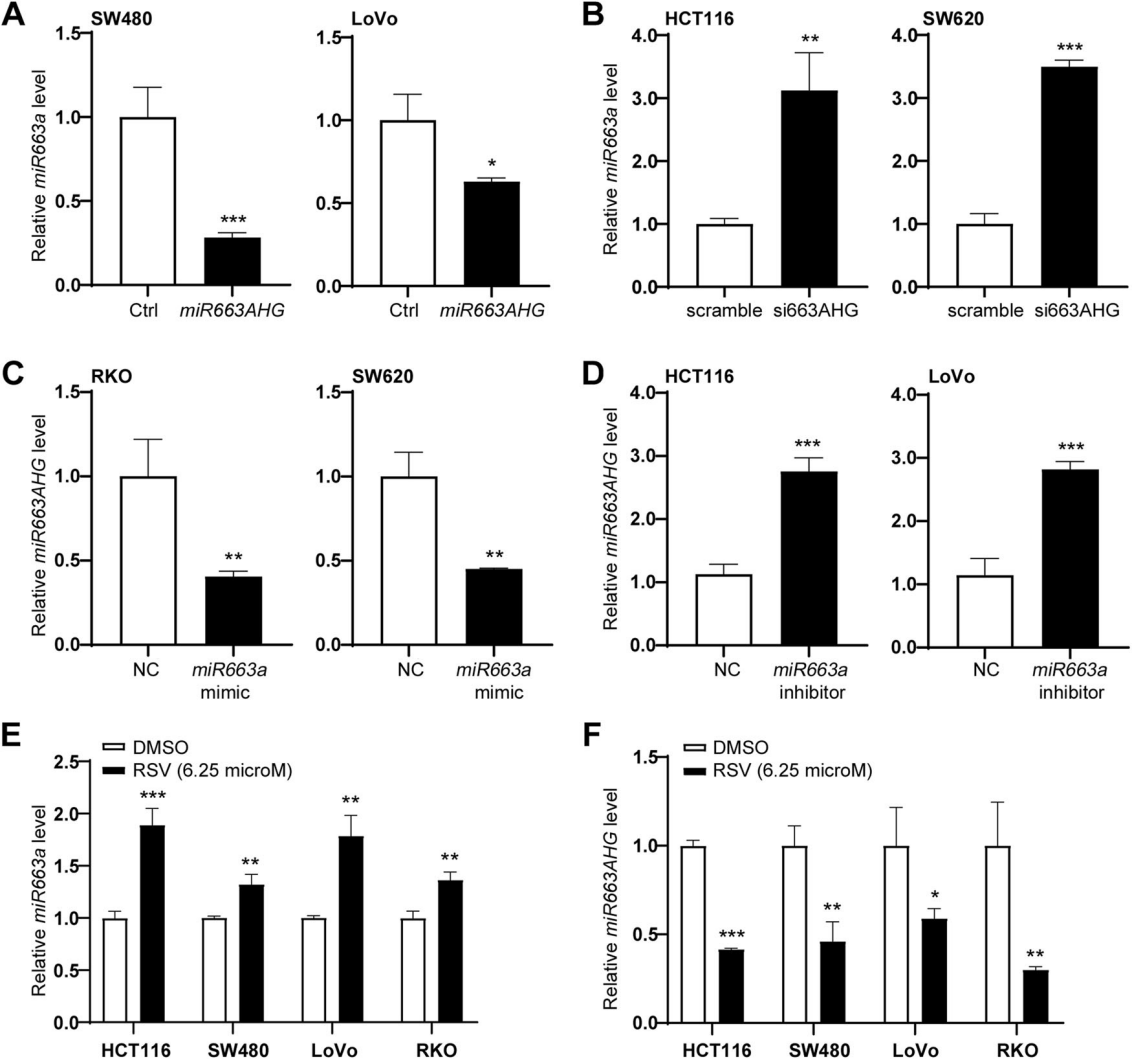
Negative feedback loop regulation between expression changes of *miR663AHG* and *miR663a* without or with resveratrol treatment. **A** Downregulation of *miR663a* by transient *miR663AHG* overexpression. **B** Upregulation of *miR663a* by siRNA knockdown of *miR663AHG*. **C**, **D** Downregulation or upregulation of *miR663AHG* by transfection of *miR663a* mimic or antisense/inhibitor. **E**, **F** Effect of resveratrol treatment on *miR663AHG* and *miR663a* expression in colon cancer cells. */**/***: *P* < 0.05/0.01/0.001 by Student’s t-test.

The *cis-*regulation between miRNAs and their host genes provides a novel layer for ncRNA-mediated gene regulation [25–27]. qRT-PCR analysis revealed that *miR663AHG* overexpression or knockdown significantly reversed endogenous *miR663a* expression in various colon cancer cell lines (Fig. 5A, B). Similarly, transfection of *miR663a* mimic or antisense/inhibitor also reversed the level of endogenous *miR663AHG* in these cell lines (Fig. 5C, D).

*miR663a* is a well-recognized miRNA involved in host responses to stress and inflammatory factors, including reactive oxygen species (ROS) [28]. Resveratrol (RSV) is a well-known antioxidant. RSV treatment increases the level of *miR663a* in various cells [29, 30]. Here, we found that RSV treatment (final concentration 6.25 μM for 12 h) not only significantly increased the *miR663a* level but also decreased the *miR663AHG* level in various colon cancer cell lines (Fig. 5E, F). These phenomena suggest that expression changes of *miR663AHG* or *miR663a*, whether induced by transfection or antioxidant treatment, could result in negative expression feedback of the *MIR663AHG* gene.

**Fig. 6:**
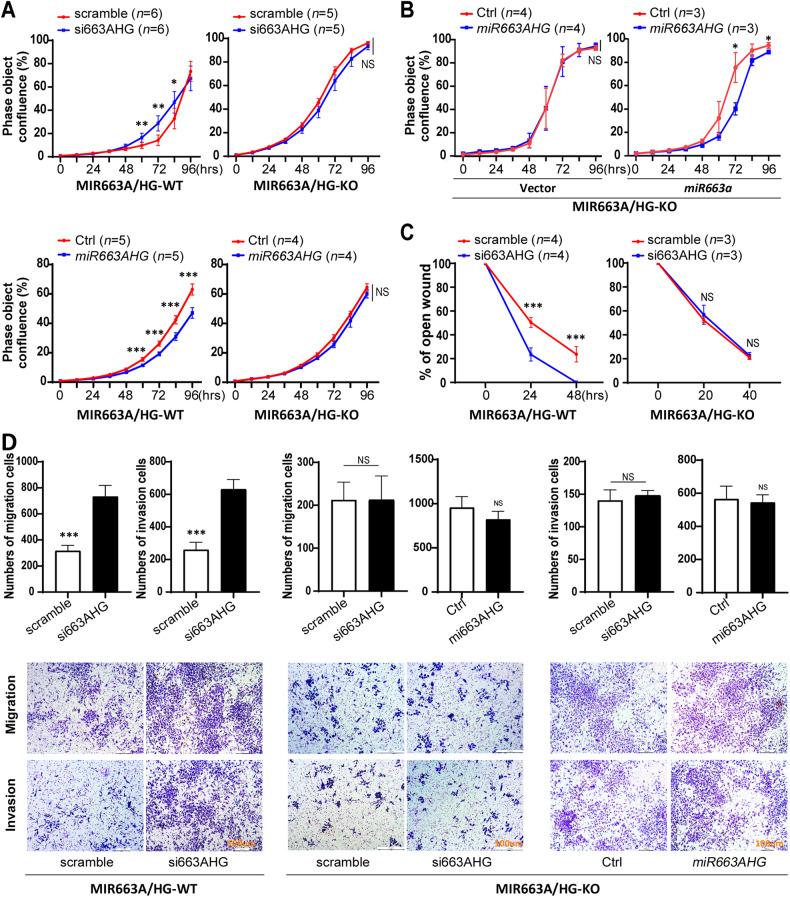
Effects of *miR663AHG* on the proliferation, migration, and invasion of MIR663A-WT and MIR663A/HG-KO RKO cells. **A** The proliferation curves of MIR663A-WT and MIR663A/HG-KO cells with and without *miR663AHG* overexpression or knockdown in long-term live cell dynamic observation. **B** Effect of *miR663AHG* overexpression on the proliferation of MIR663A/HG-KO cells with and without *miR663a* expression in the rescue experiment. **C** Effect of *miR663AHG* knockdown on the migration of MIR663A-WT and MIR663A/HG-KO cells, as determined with a wound healing assay. **D** Effect of *miR663AHG* overexpression and knockdown on the migration and invasion of MIR663A-WT and MIR663A/HG-KO cells, as determined with Transwell assays (n = 5). */**/***: *P* < 0.05/0.01/0.001 and NS, *P* > 0.05 by Student’s t-test.

To evaluate the importance of the negative feedback in *miR663AHG* function, we knocked out the 751-bp genomic sequence from the *MIR663AHG* promoter to the *pri-miR663a*-coding fragment in RKO cells with CRISPR/Cas9 (MIR663A/HG-KO; Fig. S2A–C). The level of *miRNA663AHG* expression was decreased by ~80% in MIR663A/HG-KO cells relative to wild-type control cells (MIR663A/HG-WT; Fig. S2D). While enforced *miR663AHG* expression changes significantly enhanced or inhibited the proliferation of MIR663A/HG-WT RKO cells, such effects could not be observed in MIR663A/HG-KO cells in the long observation (Fig. 6A). In the rescue experiment, the inhibitory effect of *miR663AHG* on the proliferation was restored in MIR663A/HG-KO cells transfected with the *miR663a* expression vector (Fig. 6B). Similarly, *miR663AHG* knockdown significantly enhanced the migration and invasion of MIR663A/HG-WT cells but did not affect those of MIR663A/HG-KO cells in the wound healing, transwell migration and invasion analyses (Fig. 6C, D). No effect of *miR663AHG* overexpression on the migration and invasion of MIR663A/HG-KO cells was observed. These results demonstrate that these effects of *miR663AHG* are dependent on the negative feedback of the *MIR663AHG* gene and consequent changes in *miR663a* expression.

### *miR663AHG* binds to *miR663a* and its precursors and protects *miR663a* targets

**Fig. 7:**
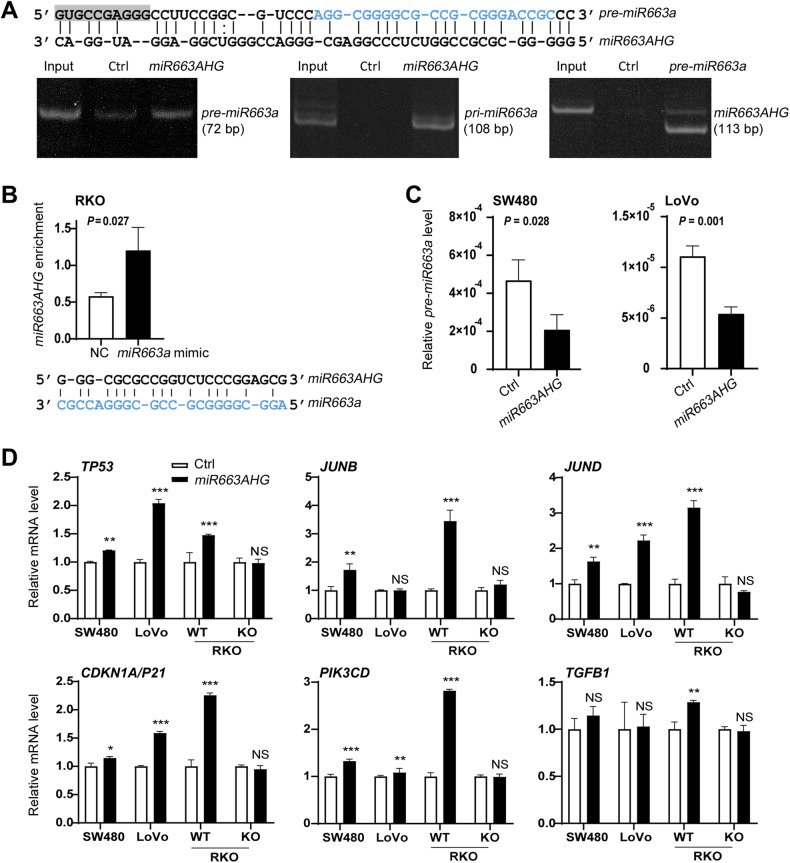
*miR663AHG* protects *miR663a* targets through *cis-*binding to *miR663a* and its precursors. **A** PAGE gel images of RNA pulldown-PCR analysis to detect *miR663AHG cis*-binding to *pre-* and *pri-miR663a*. Starbase v3.0-predicted sequences for *miR663AHG-pre-miR663a cis-*binding are illustrated on the top. Blue letters represent the sequence of mature *miR663a*. **B** The results of RNA pulldown-qRT-PCR analysis to detect the enrichment status of *miR663AHG* by biotin-labeled *miR663a* in RKO cells. Starbase v3.0-predicted sequences for *miR663AHG*-*miR663a cis-*binding are included under the chart. **C** Effect of transient *miR663AHG* overexpression on the level of *pre-miR663a* expression in colon cancer cells. **D** Effects of *miR663AHG* overexpression on the mRNA levels of *miR663a* target genes in colon cancer cells, with wild-type (WT) or knockout (KO) of *MIR663A/HG* alleles. */**/***: *P* < 0.05/0.01/0.001, NS: *P* > 0.05 by Student’s t-test.

To explore the potential underlying mechanism of the inhibitory roles of *miR663AHG* in colon cancer development, we performed bioinformatics analysis using the StarBase v3.0 database. We found that *miR663AHG* might directly bind *in cis* to the *miR663a* precursors *pre-miR663a* (and *pri-miR663a*) (Fig. S4). As expected, the results of RNA pulldown analysis showed that biotin-labeled *miR663AHG* indeed bound to *pre-miR663a* (and *pri-miR663a*) in RKO cells and that biotin-labeled *pre-miR663a* also bound to *miR663AHG* (Fig. 7A). In addition, *miR663AHG* was also significantly enriched by biotin-labeled *miR663a* in RKO cells (Fig. 7B). These results confirmed that *miR663AHG* could directly bind to *miR663a* and its precursors.

To understand the significance of *miR663AHG*-*pre-miR663a* and *miR663AHG*-*miR663a cis-*binding, we further analyzed the effects of *miR663AHG* overexpression on the *pre-miR663a* level in colon cancer SW480 and LoVo cells. Similar to *miR663a* (Fig. 5A), the level of *pre-miR663a* was significantly decreased by *miR663AHG* overexpression in these cells (Fig. 7C). These results indicate that *miR663AHG*-*pre-miR663a cis-*binding may contribute to expression changes of *miR663a* and its precursor.

In addition, we also determined the expression changes of a set of *miR663a* targets. We found that the mRNA levels of the *TP53*, *JUNB*, *JUND*, *PIK3CD*, *P21*, and *TGFB1* genes were significantly elevated by *miR663AHG* overexpression in SW480, LoVo, and MIR663A/HG-WT RKO cells. However, *miR663AHG* overexpression did not affect the mRNA levels of these targets in MIR663A/HG-KO RKO cells (Fig. 7D).

## Discussion

Primary transcripts of the *MIR663AHG* gene can be spliced into both *miR663a* and *miR663AHG*. While *miR663a* contributes to host defense responses to inflammatory factors such as reactive oxygen species (ROS) in cancer development [7, 16, 31–35], the biological function of *miR663AHG* has not been previously reported. In this study, we reported for the first time that *miR663AHG* inhibits the development and progression of human colon cancer in a *miR663a*-dependent manner.

According to the GTEx databases for 570 donors [36], three main *miR663AHG* isoforms (ENST00000608521, ENST00000608487, and ENST00000601079) are prevalently expressed in various human normal tissues with a similar expression pattern, and some rare *miR663AHG* isoforms are specifically expressed in the normal testis (Data file 1). Most *miR663AHG* isoforms are significantly downregulated in testicular germ cell tumors (TGCT) and acute myeloid leukemia (LAML), according to the results of The Gene Expression Profiling Interactive Analysis (GEPIA2) using Cancer Genome Atlas (TCGA) RNA-seq datasets (Figure S5) [37, 38]. Using a sensitive and quantitative transcript-specific RT-PCR assay, we found that *miR663AHG* (ENST00000608521) was significantly downregulated in colon cancer tissues and associated with colon cancer metastasis. Our in vitro and in vivo experiments consistently support the hypothesis that *miR663AHG* functions as a tumor suppressor. The roles of other *miR663AHG* isoforms in cancer development are worthy of further study.

The functions of most biological macromolecules depend on their subcellular distributions. In our RNA-FISH experiment, a set of fluorescently labeled RNA probes targeting various *miR663AHG* isoforms were used. We found that *miR663AHG* was distributed in the cytoplasm and nucleus of colon cancer cell lines. No *miR663AHG* signal was detected in LoVo cells with a low, if any, baseline level of *miR663AHG* expression, indicating high accuracy of the RNA-FISH assay. However, cytoplasmic *miR663AHG* signals were detected in SW480 cells with a low level of baseline *miR663AHG* expression, suggesting the possibility of other *miR663AHG* isoforms in these cells, which may not be covered by the *miR663AHG*/ENST00000608521-specific RT-PCR primer set used in the present study. To confirm this, we mined CCLE RNA-seq datasets and found that the expression levels of two *miR663AHG* isoforms ENST00000615682 and ENST00000665857, which are most frequently expressed (~10%) in cancer cell lines (*n* = 1406), are detectable in SW480 cells (Data file 2) [23]. The RT-PCR primer set indeed does not cover ENST00000615682 and ENST00000665857. This may account for the difference in the expression level and subcellular location of *miR663AHG* between SW480 and HCT116 or Caco2 cells.

It is well recognized that pre-miRNAs are nuclear-cytoplasmic transportation forms, and mature miRNAs are mainly distributed in the cytoplasm. That *miR663AHG* is located in both the nucleus and cytoplasm is consistent with the observation of *miR663AHG cis-*binding to both *pre-miR663a* and *miR663a*. Interestingly, we found that *miR663AHG* could bind to *pri-miR663a*, suggesting an additional regulatory machine in the nucleus. *miR663AHG* may regulate the functions of *miR663a* at multiple levels.

Our and others’ works indicate that *miR663a* inhibits the development and metastasis of colon cancer and that the *miR663a* target genes *MALAT1* and *TTC22* promote colon cancer metastasis [7, 16, 39, 40]. Here, we further found that *miR663AHG* could inhibit the proliferation, migration/invasion of colon cancer cells in vitro and the growth and lung metastasis of colon cancer cells in vivo.

As described above, while *miR663AHG* prevents *miR663a* targets from degradation, both *miR663AHG* and *miR663a* inhibit the development and metastasis of colon cancer. In addition, the expression levels of *miR663AHG* and *miR663a* are positively correlated with each other not only in colon tissues from cancer patients but also in ovarian cancer tissues. Notably, disruption of the negative feedback of the *MIR663AHG* gene entirely abolished the effects of *miR663AHG* on the biological behaviors of cancer cells. These phenomena suggest that the functions of endogenous *miR663AHG* and *miR663a* may be intrinsically connected: they form the *miR663AHG-miR663a* axis and play the same role in maintaining homeostasis of defense responses of host cells to inflammatory factors and cancer development.

Mechanistically, we found that alterations of *miR663AHG* (or *miR663a*) expression, whether induced by expression vectors or the antioxidant resveratrol treatment, could reverse alterations of endogenous *miR663a* (or *miR663AHG*) expression, demonstrating the presence of a negative feedback loop of the expression of the *MIR663AHG* gene. However, both *miR663AHG* and miR663a are spliced from the same primary transcript. The abovementioned positive correlation of *miR663AHG* and *miR663a* expression in human tissues suggests that the transcriptional activity of the *MIR663AHG* gene should be a dominant factor in determining the expression levels of *miR663AHG* and *miR663a* in human cells, although their exact expression levels and biological functions are inversely affected by each other.

In conclusion, our study demonstrates that lncRNA *miR663AHG* binds to *miR663a* and its precursor *pre-miR663a* and induces the negative feedback of *miR663a* expression. *miR663AHG* may play an important role in the host defense responses to inflammation and in the inhibition of colon cancer development through *cis-*binding to *miR663a* and its precursors.

## Methods

### Clinical sample collection

Colon cancer and paired normal surgical margin tissue samples (SMs, >5 cm from cancer lesions) were collected from 119 patients (Table S1) at Peking University Cancer Hospital from 2004 to 2011 and stored at −80 °C. These patients were enrolled in our previous studies [7, 16]. This study complied with the 1964 Declaration of Helsinki and was approved by the Institutional Review Board of the Peking University Cancer Hospital. All patients provided informed consent before sample collection.

### Cell lines and culture

The colon cancer cell lines HCT116 and SW480 were kindly provided by Professor Yuanjia Chen at Peking Union Medical College Hospital; SW620 was kindly provided by Professor Chengchao Shou at Peking University Cancer Hospital; RKO was kindly provided by Dr. Guoren Deng at the University of California; Caco2 was purchased from National Infrastructure of Cell Line Resource (Beijing, China); and LoVo was purchased from the American Type Culture Collection (ATCC, Manassas, USA). The human cell line HEK293FT was kindly provided by Professor Yasuhito Yuasa at Tokyo Medical and Dental University. HCT116, SW480, RKO, and SW620 cells were cultured in RPMI-1640 medium containing 10% FBS. HEK293FT cells were cultured in DMEM with 10% FBS. Caco2 cells were cultured in MEM with 10% FBS and 1% NEAA (Invitrogen, Gibco, USA), and LoVo cells were cultured in F12K medium with 10% FBS. All media were supplemented with 100 U/mL penicillin/streptomycin (Life Technologies, Carlsbad, CA, USA). All cell lines were tested and authenticated by Beijing JianLian Gene Technology Co. before they were used. Short tandem repeat (STR) patterns were analyzed using the Goldeneye 20A STR Identifier PCR Amplification Kit.

### Plasmid construction and transfection

Full-length *miR663AHG* (ENST00000608521.6) was synthesized by GenScript (Nanjing, China) and then inserted into the pHBLV-CMV-MCS-EF1-ZsGreen-T2A-puro lentiviral expression vector (Hanbio, Shanghai, China). The empty control and *miR663AHG* expression vectors were generated with a lentiviral packaging kit (Syngentech Co., Ltd., Beijing, China) according to the manufacturer’s manual in HEK293FT cells [6]. The lentivirus particle-containing culture medium was collected 48 h after transfection, filtered with a 0.45-μm filter, and added to colon cancer cells. The stably infected cells were selected for three days with 1 μg/mL puromycin (Sigma, St. Louis, MO, USA). X-tremeGENE HP DNA Transfection Reagent (Roche, Mannheim, Germany) was used in transient transfection with the *miR663AHG* expression vector following the manufacturer’s instructions. Transfection efficiency was monitored with the density of the ZsGreen tag integrated within these vectors and RT-PCR for target genes in the cells.

*miR663a* mimic and antisense/inhibitor were purchased from RiboBio Co., Ltd. (Guangzhou, China) [7, 16]. siRNAs against *miR663AHG* (si663AHG#1: sense 5’-gaggugcuuugccucugaatt-3’ and antisense 5’-uucagaggcaaagcaccuctt-3’; si663AHG#2: sense 5’-gcaugcaaugggcaaucuatt-3’ and antisense 5’-uagauugcccauugcaugctt-3’) were synthesized by Genepharma Co. (Shanghai, China). A mixture of siR663AHG #1 and #2 was used to knock down *miR663AHG* expression (siR663AHG). X-tremeGENE siRNA Transfection Reagent (Roche, Mannheim, Germany) was used in transfection with these siRNAs above. Transfection efficiency was verified using RT-PCR for *miR663a* or *miR663AHG* in the cells.

### RNA extraction and quantitative RT-PCR assays (qRT-PCR)

Total RNA was isolated using TRIzol Reagent (ZYMO Research, Beijing, China) according to the manufacturer’s instructions. First-strand cDNA was generated using the TransScript First-Strand cDNA Synthesis Kit (TransGen Biotech, Beijing, China). Exon-exon qRT-PCR was performed using FastStart Universal SYBR Green Master Mix (Roche, Mannheim, Germany). U6 small nuclear RNA was used as an internal control for *miR663a*; the level of *miR663AHG* was normalized to that of *GAPDH* (for cultured cells) or *Alu* (for tissues). Similarly, the levels of *TP53*, *JUNB*, *JUND*, *PIK3CD*, *P21*, and *TGFB1* mRNAs in cell lines were normalized to that of *GAPDH*. The relative mRNA levels further normalized to the control were further calculated using the typical 2^-ΔΔCT^ method [7]. The relative level of *pre-miR663a* was calculated according to the formula [41]: *pre-miR663a* = 2^-CT(*pri-miR663a*+*pre-miR663a*)^ − 2^-CT(*pri-miR663a*)^. Sequences of these PCR primers are listed in Table S2.

### RNA-FISH assay

Fluorescence-conjugated *miR663AHG* probes for *miR663AHG* were purchased from RiboBio Co., Ltd (Guangzhou, China). According to the manufacturer’s instructions, cells were pretreated with 4% paraformaldehyde, hybridized with *miR663AHG* probes labeled with Cy3, and stained with DAPI using the Ribo Fluorescent In Situ Hybridization kit (RiboBio Co., Ltd., Guangzhou, China). Probes for *18S rRNA* and *U6 RNA* were used as cytoplasmic and nuclear RNA controls. Images were obtained with a Leica SP5 Laser Scanning Confocal Microscope (Leica, Germany).

### Cell proliferation assay using IncuCyte

Long-term dynamic observation assays were used to detect cell proliferation [6]. Briefly, all cells were seeded in 96-well plates at a density of 2 × 10^3^ cells per well. To measure proliferation, cells were photographed every 12 h in the long-term dynamic observation platform (IncuCyte, Essen, MI, USA) for at least 96 h. IncuCyte ZOOM software (Essen, Ann Arbor, MI, USA) was used to analyze cell confluence.

### Wound healing assay

All cells were seeded in 6-well plates at 95–100% confluence before the experiment. A pipette tip was used to scratch the cell monolayer gently. After washing with 1 × PBS twice, the cells were cultured with RPMI-1640 medium with 1% FBS. Images of wound healing were captured at different times by a microscope (Nikon, Japan).

### Transwell assays

For the migration assay, cells (2 × 10^4^ cells per chamber) were separately seeded in the upper chambers (8 μm pores; Corning Inc., Corning, NY). For the invasion assay, the upper chambers were precoated with Matrigel (BD Biosciences, Franklin Lakes, NJ, USA). Then, cells (4 × 10^4^ cells per chamber) were separately seeded in the upper chambers with the same aperture. All cells were resuspended in 180 μL of serum-free RPMI-1640 medium before seeding. After a 24–48 h incubation, the chambers were fixed with 4% paraformaldehyde for 30 min and then stained with 0.1% crystal violet. Nonmigrated/noninvasive cells on the upper surface of the insert were wiped with a cotton swab. Images of migrating and invading cells were captured using a microscope (Leica DMI4000B, Milton Keynes, Bucks, UK).

### Animal experiments [42]

Six-week-old female BALB/c mice (purchased from Beijing Huafukang Biotech) were used for the subcutaneous xenograft experiment. RKO cells were resuspended in 0.10 mL 1× PBS (1 × 10^7^ cells/mL) and inoculated subcutaneously into the bilateral inguinal region of mice (6 mice per group, 1 × 10^6^ cells per injection). Mice were sacrificed on the 21st inoculation day. All xenografts were separated, weighed, and photographed. For the experimental lung metastatic model, LoVo cells were resuspended in 0.10 mL PBS (1 × 10^7^ cells/mL) and then injected into the tail vein of NOD/SCID mice (7 mice per group, 1 × 10^6^ cells per injection). The mice were sacrificed eight weeks post-injection. All lung tissues were excised, weighed, and photographed. No randomization was used and no blinding was done. This study was approved by the Institute’s animal ethics committee.

### Knockout of the genomic *MIR663A/HG* sequence by CRISPR/Cas9 [43, 44]

A dual gRNA approach was used to knock out the DNA sequence from the *MIR663AHG* promoter to *miR663a* by the CRISPR/Cas9 system. The oligonucleotides used for single guide RNA (sgRNA) construction were individually designed for the target sequence (Fig. S2 and Table S2). They were synthesized by Thermo Scientific, Inc. (Rockford, IL, USA). These gRNAs were cloned into the PX458 vector (Plasmid #48138, Addgene, Inc.) and transfected into RKO cells. Then, a flow sorting assay was performed by green fluorescence with a FACSCalibur flow cytometer (BD Biosciences, Franklin Lakes, USA) 48 h post-transfection. Two weeks later, monoclonal cells in good growth conditions were selected, and the knockout status of the target sequence in subclones was identified by PCR (Fig. S2B). The results of PCR sequencing revealed that a 751-bp genomic sequence (including the *MIR663AHG* promoter, exon-1, and *pri-miR663a-*encoding fragment) was knocked out (MIR663A/HG-KO) (Fig. S2C, gray line polygon).

### RNA pulldown assay [6]

The full-length sequences of *miR663AHG* and *pre-miR663a* were amplified from the above vector and genomic DNA with a primer set (forward 5’-gtcccacggtgggggcg-3’ and reverse 5’-agttactttaaggctttatttgt-3’) and a primer set (forward 5’-ccttccggcgtcccagg-3’ and reverse 5’-catggccgggccacca-3’) and inserted into the pGEM-T easy vector [45]. These vectors were cleaved using the restriction endonuclease *SpeI*, and the in vitro transcription experiment followed the instructions of the Riboprobe In Vitro Transcription Systems (Promega, Madison, WI, USA). Then, the transcribed *miR663AHG* or *pre-miR663a* RNA products were labeled by biotin in vitro using the Pierce RNA 3′ End Desthiobiotinylation Kit (Thermo Scientific, Rockford, IL, USA). The biotin-labeled products were bound to streptavidin magnetic beads and incubated with separated lysates from MIR663A/HG-WT or MIR663A/HG-KO RKO cells according to the instructions of the Pierce Magnetic RNA-Protein Pull-Down Kit (Thermo Scientific, Rockford, IL, USA). RNA from the pulldown components was separated, reverse transcribed, and analyzed with quantitative PCR assays. The PCR products were analyzed using 10% nondenaturing PAGE gel electrophoresis.

### The download of Cancer Cell Line Encyclopedia (CCLE) and The Cancer Genome Atlas (TCGA) datasets

*MIR663AHG* promoter DNA methylation data (1 kb upstream of the promoter) were extracted from the CCLE DNA methylation RRBS data file (promoter methylation -1 kb upstream of the promoter). These cell lines were subclassified into three methylation groups (methylation high, moderate, and low) with equal size. *miR663AHG* expression data were extracted from the RNA-seq data file (CCLE RNAseq gene expression data for 1019 cell lines, RPKM); *miR663a* expression data were extracted from the RNA-seq miRNA expression data file (CCLE miRNA expression data). These datasets [23] were downloaded from the CCLE official website (https://sites.broadinstitute.org/ccle/datasets). The expression levels of *miR663AHG* and *miR663A* were extracted from miRNA and lncRNA array datasets for 352 ovarian carcinoma samples in a previous study (Data file 3) [24].

### Statistical analyses

Data analysis and processing software were mainly GraphPad Prism 5.0 software (San Diego, CA, USA) and IBM SPSS 23.0 software (SPSS Inc., Chicago, IL, USA). The Kolmogorov-Smirnov test or Shapiro-Wilk test was used to test whether the data conformed to a normal distribution. Student’s t-test and Pearson correlation analysis were used for data conforming to a normal distribution, while the Mann–Whitney U-test and Spearman correlation analysis were used for nonnormally distributed data. Survival analysis of patients with colon cancer was performed using the Kaplan-Meier method. Cox risk proportional regression models were used in the univariate and multifactor survival analyses. *P* < 0.05 (two-sided) was considered a statistically significant difference. All biological experiments were repeated one or two times.

## Supplementary information

Figure S1-S5

Supplemental Table S1-S3

Data files 1 - 3

## Data Availability

All additional datasets were presented in Data files 1–3.
