# Supplementary material for: LncRNA miR663AHG represses the development of colon cancer in a miR663a-dependent manner
Source: Cell Death Discov. 2023 Jul 3;9:220. doi: 10.1038/s41420-023-01510-1 (PMC10317963; doi:10.1038/s41420-023-01510-1)
Supplement: Supplementary file 1 — Figure S1-S5 [file 41420_2023_1510_MOESM1_ESM.pdf]

Supplemental Figures

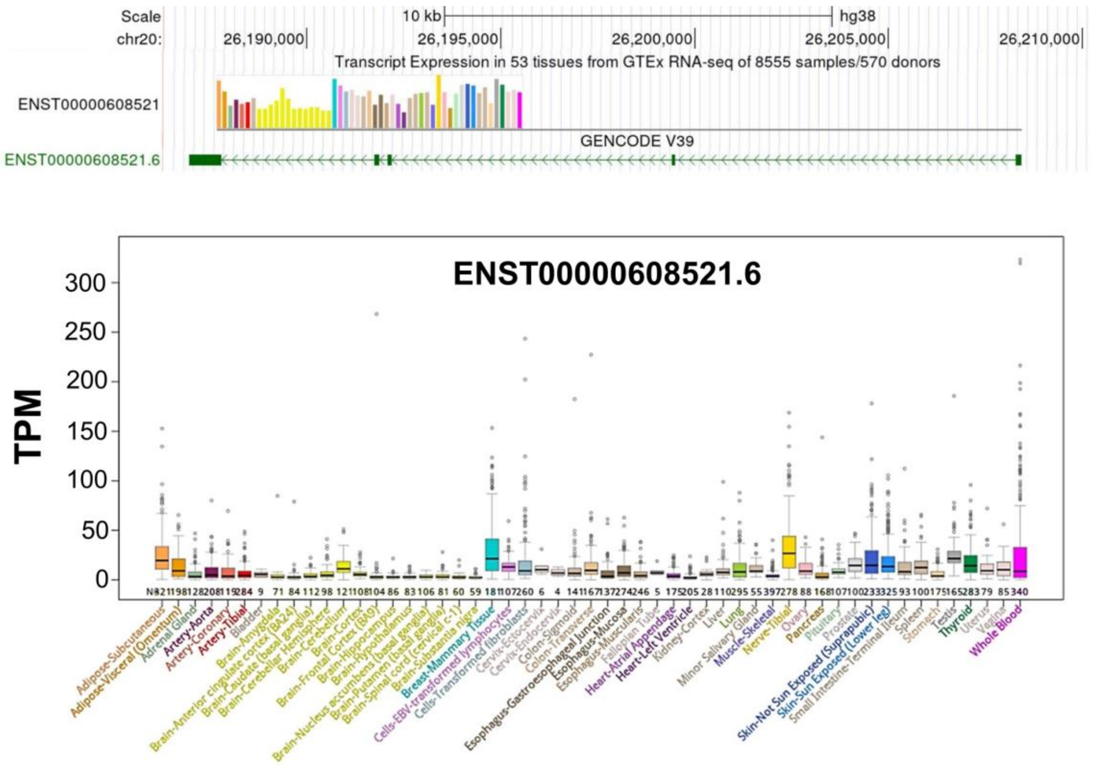

Figure S1. The prevalence of *miR663AHG* ENST00000608521 transcript in normal human tissues according to the GTEx datasets [41]. The locations of the exon-exon RT-PCR primer set for *miR663AHG* are illustrated. The exact sequences are listed in Table S2. These images were adapted from graphic views downloaded from the UCSC Genome Browser website [http://genome.ucsc.edu].

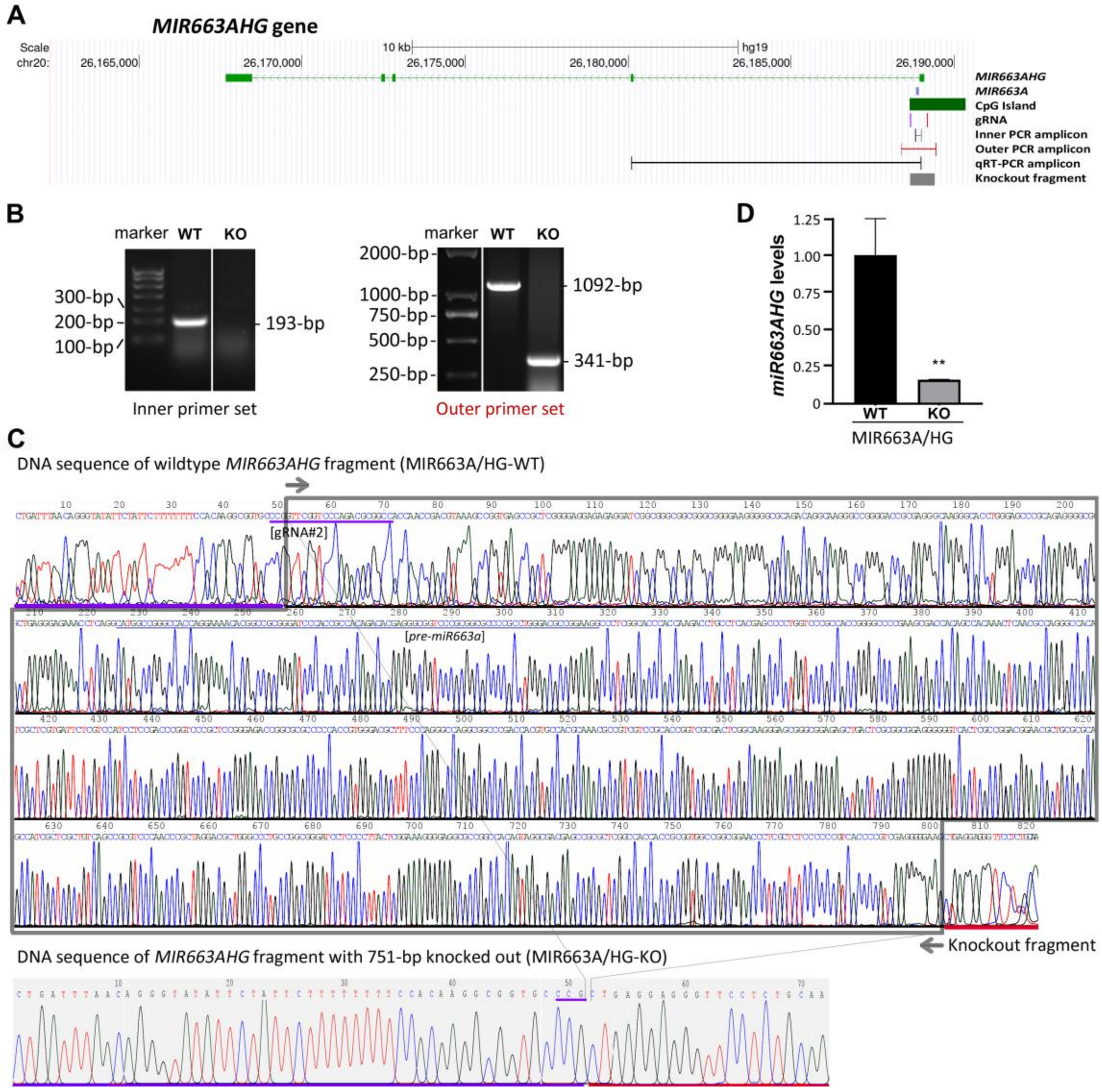

Figure S2. CRISPR/Cas9 knockout (KO) of the genomic *MIR663A* sequence. (**A**) Schematic diagram of two guide RNAs (gRNA #1 and #2) matching sequences of the *MIR663AHG* gene in RKO cells. Locations of the primer sets for RT-PCR, outer andinner amplicons, and the knockout fragment are illustrated. Their exact oligo sequences are listed in Table S2. (**B**) The PCR results of screening the KO status of the genomic sequence of the *MIR663AHG* gene. WT and KO, RKO cells without and with knockout of the 751-bp genomic *MIR663AHG* fragment; (**C**) Graphic view of sequences of PCR products (by Outer primer set) amplified from genomic DNA of the *MIR663A/HG*-KO RKO clone and WT/mock control. Black underlined sequence, the 751-bp Cas9-knocked out fragment, including the *MIR663AHG* promoter and exon-1, and *pre-miR663a* (93-bp); violet and red underlined sequences, remaining genomic sequences flanking the knockout fragment; (**D**) The level of *miR663AHG* expression in MIR663A/HG-KO and -WT RKO cells.

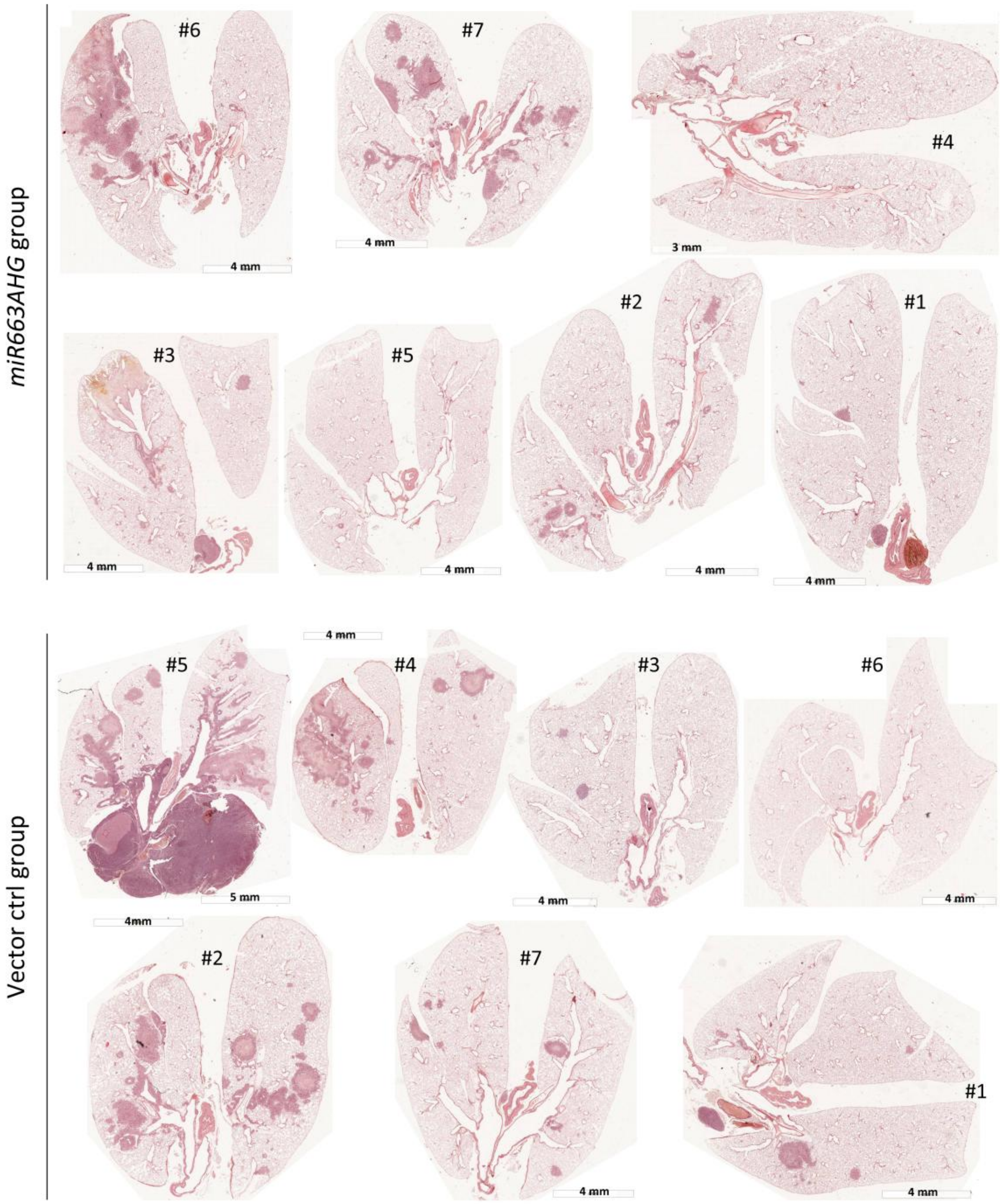

Figure S3. Photos of lung sections for mice tail-vein-injected with LoVo cells with and without *miR663AHG* overexpression.

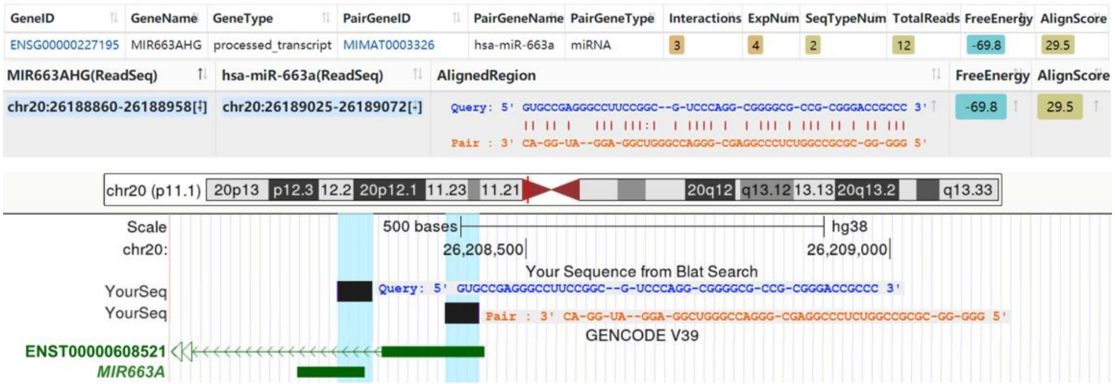

Figure S4. Potential *miR663AHG-pre-miR663a cis*-binding sequences were predicted by StarBase v3.0 (top) and genomic locations of these sequences in the human genome.

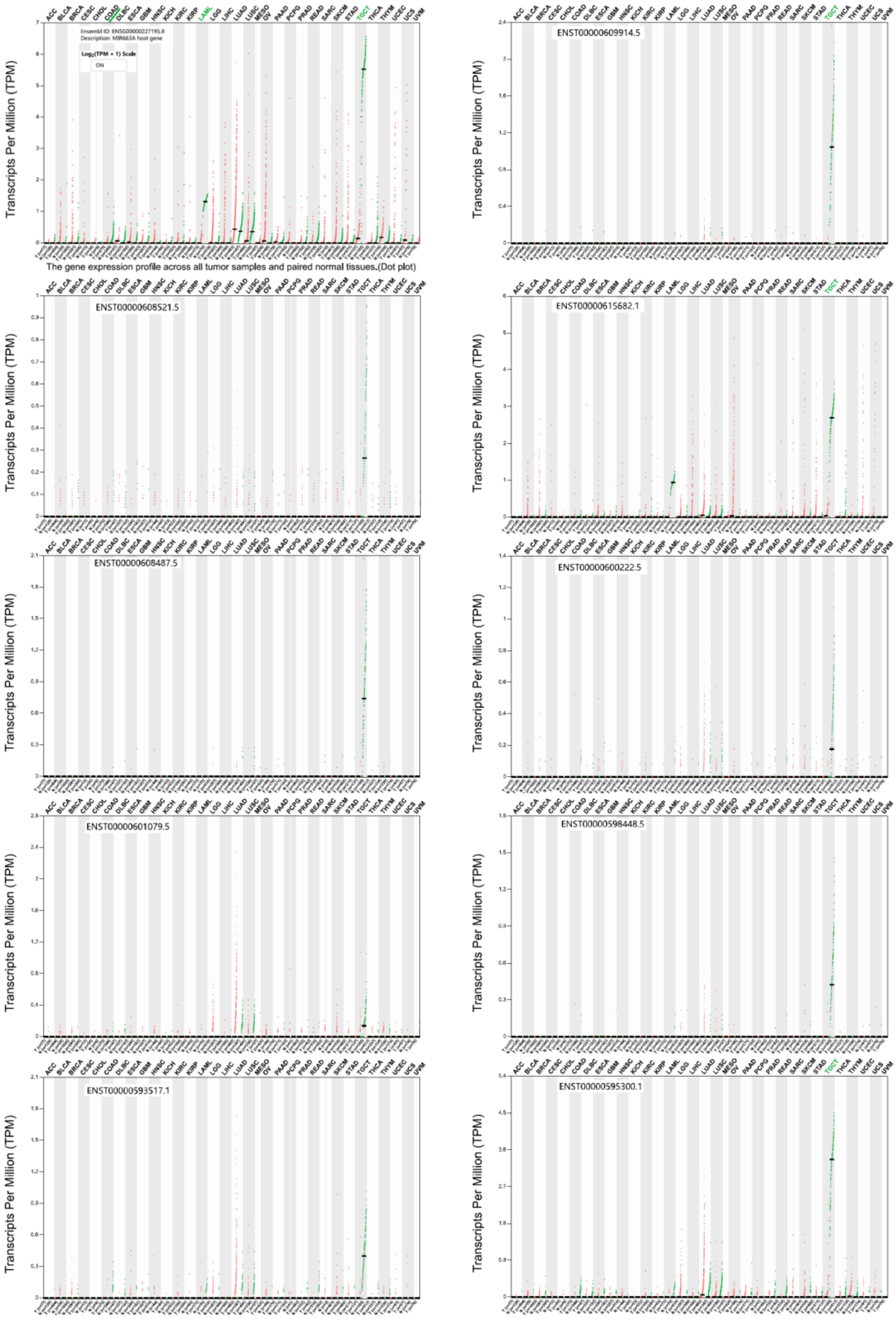

Figure S5. Comparisons of the expression levels of total *miR66AHG* (top left chart) and its main transcripts in all tumor samples and paired normal samples according to the TCGA RNA-seq datasets in the results of the online GEPIA2 analysis [42, 43].
