# Supplementary material for: LncRNA miR663AHG represses the development of colon cancer in a miR663a-dependent manner
Source: Cell Death Discov. 2023 Jul 3;9:220. doi: 10.1038/s41420-023-01510-1 (PMC10317963; doi:10.1038/s41420-023-01510-1)
Supplement: Supplementary file 2 — Supplemental Table S1-S3 [file 41420_2023_1510_MOESM2_ESM.docx]

**Table S1.** Comparison of the level of lncRNA *miR663AHG* between colon cancer subgroups with different clinicopathological characteristics

| Characteristic | *n* | Relative level of lncRNA *miR663AHG* in colon cancer ^a^ | *P-*value ^b^ |
| --- | --- | --- | --- |
| Age | | | |
| <50 | 19 | 1.92 (0.59-4.54) | 0.121 |
| ≥50 | 100 | 4.03 (1.03-8.96) |  |
| Gender | | | |
| male | 68 | 4.17 (0.95-7.83) | 0.924 |
| female | 51 | 2.76 (0.99-9.00) |  |
| pTNM stage | | | |
| I-II | 59 | 4.66 (1.21-9.84) | 0.021 |
| III-IV | 58 | 1.92 (0.86-6.45) |  |
| NA | 2 | / |  |
| T stage | | | |
| T1-T2 | 6 | 11.4 (2.55-26.1) | 0.236 |
| T3-T4 | 111 | 2.86 (0.97-7.78) |  |
| NA | 2 | / |  |
| N stage | | | |
| N0 | 60 | 4.57 (0.98-9.53) | 0.041 |
| N1-N2 | 57 | 1.92 (0.92-6.46) |  |
| NA | 2 | / |  |
| M stage | | | |
| M0 | 91 | 4.12 (0.93-9.00) | 0.176 |
| M1 | 28 | 2.02 (1.14-6.25) |  |
| Differentiation | | | |
| well | 9 | 2.86 (2.04-9.01) | 0.049 |
| moderate | 82 | 3.06 (0.74-7.79) |  |
| poor | 25 | 4.11 (1.59-8.99) |  |
| NA | 3 | / |  |

^a^ Alu-normalized relative lncRNA level (×10^−5^), the values are presented as median and ranges (25% - 75% percentile); ^b^ *P* < 0.05 indicates statistically significant.

**Table S2.** Sequences of primers and guide RNA (gRNA)

| Assays | Oligo name | Oligo sequence(5’ - 3’) |
| --- | --- | --- |
| qRT-PCR Primers | *MIR663AHG* FP | tggctgtggtcgcttcg |
|  | *MIR663AHG* RP | tatgggctgaactgtgctcc |
|  | *pri-miR663a* FP | ggctcgtgaggcaggtctt |
|  | *pre-miR663a* FP | ttccggcgtcccaggc |
|  | *pri*/*pre-miR663a* RP | aaacacggccgcgggat |
|  | *JUND* FP | gtctacgcgaacctgagcagcta |
|  | *JUND* RP | ctcgtccttgagcgcagccaggc |
|  | *JUNB* FP | atggaacagcccttctaccacg |
|  | *JUNB* RP | aggctcggtttcaggagtttg |
|  | *P53* FP | cccaagcaatggatgatttga |
|  | *P53* RP | ggcattctgggagcttcatct |
|  | *PIK3CD* FP | tcaactcacagatcagcctcc |
|  | *PIK3CD* RP | ttcacttctgggtcgcacaag |
|  | *TGFB1* FP | aaggacctcggctggaagtg |
|  | *TGFB1* RP | cccgggttatgctggttgta |
|  | *P21* FP | gcagaccagcatgacagattt |
|  | *P21* RP | ggattagggcttcctcttgga |
|  | *ALU* FP | gaggctgaggcaggagaatcg |
|  | *ALU* RP | gtcgcccaggctggagtg |
| RT-PCR Out-primer | *MIR663A* FP-1 | tctccgttatggtagcgctg |
|  | *MIR663A* RP-1 | cttgactccaaagctgcacc |
| RT-PCR Inner-primer | *MIR663A* FP-2 | cgttgagtttgtggctgtg |
|  | *MIR663A* RP-2 | agggagaaacctcaggcat |
| CRISPR/Cas9 gRNA | PX458-sgRNA#1 S | caccgagctgactcgcggcggaggg |
|  | PX458-sgRNA#1 AS | aaacccctccgccgcgagtcagctc |
|  | PX458-sgRNA#2 S | caccggccgcgtctgggaccgaac |
|  | PX458-sgRNA#2 AS | aaacgttcggtcccagacgcggcc |

| Variants | Univariate analysis | | Multivariate analysis | |
| --- | --- | --- | --- | --- |
|  | HR (95% CI)* | *P-*value | HR (95% CI) | *P-*value |
| Ages  (≥50 vs. <50) | 1.016 (0.992-1.040) | 0.194 | 1.022 (0.994-1.052) | 0.120 |
| Gender (male vs. female) | 0.813 (0.455-1.454) | 0.486 | 0.885 (0.488-1.605) | 0.687 |
| Location invasion (T_3-4_ vs. T_1-2_) | 1.514 (0.367-6.250) | 0.566 |  |  |
| Lymph metastasis (N_0_ vs. N_1-2_) | 0.035 (0.011-0.114) | <0.001 | 0.046 (0.014-0.159) | <0.001 |
| Distant metastasis (M_1_ vs. M_0_) | 8.766 (4.757-16.155) | <0.001 | 3.037 (1.578-5.845) | 0.001 |
| pTNM stage  (III&IV vs. I&II) | 27.168 (8.379-88.092) | <0.001 |  |  |
| Differentiation (moderate&poor vs. well) | 1.956 (0.473-8.086) | 0.354 |  |  |
| *miR663AHG* (expression low vs. expression high) | 2.026 (1.113-3.689) | 0.021 | 0.779 (0.405-1.498) | 0.455 |

**Table S3.** Univariate and multivariate analyses of the relationship between overall survival of patients with colon cancer with different *miR663AHG* expression levels

*HR, Hazard ratio; CI, confidence interval
